# MatrixCatch - a novel tool for the recognition of composite regulatory elements in promoters

**DOI:** 10.1186/1471-2105-14-241

**Published:** 2013-08-08

**Authors:** Igor V Deyneko, Alexander E Kel, Olga V Kel-Margoulis, Elena V Deineko, Edgar Wingender, Siegfried Weiss

**Affiliations:** 1 Department of Molecular Immunology, Helmholtz Centre for Infection Research, Braunschweig, Germany; 2 GeneXplain GmbH, Wolfenbüttel, Germany; 3 Institute of Chemical Biology and Fundamental Medicine SB RAS, Novosibirsk, Russia; 4 Laboratory of Plant Bioengineering, Institute of Cytology and Genetics SB RAS, Novosibirsk, Russia; 5 Institute of Bioinformatics, University Medical Centre Göttingen, Göttingen, Germany

## Abstract

**Background:**

Accurate recognition of regulatory elements in promoters is an essential prerequisite for understanding the mechanisms of gene regulation at the level of transcription. Composite regulatory elements represent a particular type of such transcriptional regulatory elements consisting of pairs of individual DNA motifs. In contrast to the present approach, most available recognition techniques are based purely on statistical evaluation of the occurrence of single motifs. Such methods are limited in application, since the accuracy of recognition is greatly dependent on the size and quality of the sequence dataset. Methods that exploit available knowledge and have broad applicability are evidently needed.

**Results:**

We developed a novel method to identify composite regulatory elements in promoters using a library of known examples. In depth investigation of regularities encoded in known composite elements allowed us to introduce a new characteristic measure and to improve the specificity compared with other methods. Tests on an established benchmark and real genomic data show that our method outperforms other available methods based either on known examples or statistical evaluations. In addition to better recognition, a practical advantage of this method is first the ability to detect a high number of different types of composite elements, and second direct biological interpretation of the identified results. The program is available at http://gnaweb.helmholtz-hzi.de/cgi-bin/MCatch/MatrixCatch.pl and includes an option to extend the provided library by user supplied data.

**Conclusions:**

The novel algorithm for the identification of composite regulatory elements presented in this paper was proved to be superior to existing methods. Its application to tissue specific promoters identified several highly specific composite elements with relevance to their biological function. This approach together with other methods will further advance the understanding of transcriptional regulation of genes.

## Background

Deciphering the mechanisms of transcriptional regulation of gene expression is one of the key problems biologists are facing. It is widely accepted to date that genes especially, in higher eukaryotes are regulated by a combination of transcription factors (TFs) bound to their cognate DNA sites, rather than by a single factor. Therefore, an extensive research is conducted on combinatorial interactions of protein factors and their DNA binding sites (BSs) with respect to transcriptional activity of affected genes. The majority of present methods evaluate the statistical properties of motif pairs (for review see [1]) or multiple combinations of motifs [2]. Some methods use comparisons with existing examples of motif combinations as a basis for recognition [3-6].

The minimal functional unit, which can provide combinatorial regulation, is a composite element (CE). Structurally a CE consists of two closely located BSs for distinct transcription factors (TFs). But functionally CEs are considered as single elements, since its regulatory function are qualitatively different from regulation effects of either individual BSs [7,8]. Function, structure and primary sequence of CEs are studied in a number of different experiments, in particular, to confirm protein-protein interactions and cooperative binding to DNA, as well as effects on transcriptional regulation. Such data on CEs can be found in databases such as TRANSCompel [9].

The major problem in developing general recognition methods for CEs lies in the extremely limited number of experimentally defined CEs. For particular types of CEs some *ad hoc* methods have been suggested [3-5]. However, the method, which can identify many types of CEs [6] shows relatively poor recognition characteristics.

The basic idea of the current method is to complement existing knowledge on experimentally identified and functionally described CEs by data available for single BSs constituting the CEs. We demonstrate that such an integrative approach is able to model the heterogeneity of CEs, which results in good recognition characteristics of the method. We also show that the existing variety of CEs is in no way a limiting factor to the method applicability. Quite the contrary, MatrixCatch with the provided library outperformed all statistical methods, that to date attract excessive attention of bioinformatics community. Elements of crowdsourcing were implemented in the website to allow further extension the existed CE library.

## Methods

### Matrix model of CE

The idea behind MatrixCatch is to complement the lack of knowledge on sequence variation of each DNA BS in CEs by recruiting data collected for respective BSs separately from each other. Such information is compiled in position weight matrices (PWMs). Each CE will serve as a template for a model, which consists of two PWMs, as well as their minimal scores, relative orientation and distance. Thus, PWMs, which are built using many single BSs, define sequence variability of BSs in the CE. Minimal scores for PWMs, orientation and distance between PWMs are determined by the CE itself.

### Building the CE model

First, PWMs related to the first binding TF are selected from the entire TRANSFAC library (in case there are several). Here and further we call the “first” and “second” BS in a CE model in accordance to the database annotation. Second, PWM scores are calculated for both orientations at the position of the first annotated BS in CE for all selected PWMs. Third, the combination of PWM, its score and its orientation, which delivers the lowest prediction rate on random sequences, is selected. Often, but not always, it is the PWM with the highest score. This score becomes the minimal required PWM score S_*m1*_ in the model for the first BS. After repeating the same three steps for the second BS, all the parameters of the CE model are identified: PWM_*1*_, PWM_*2*_, their orientations, minimal scores S_*m1*_, S_*m2*_ and in-between distance *D*_*m*_.

On this basis, we build 265 matrix models for all CEs collected in the TRANSCompel database. To search for potential CE, MatrixCatch will test these models on a DNA sequence. To be able to reveal “non perfect matches”, model parameters like PWM scores (S_*m1*_, S_*m2*_) and distance (*D*_*m*_) should be relaxed. To increase the specificity of the search we introduced a “composite score” (CS). As will be showed later, this composite score provides higher recognition accuracy in comparison to existing methods.

### Dependence between binding sites in CEs

**Figure 1:**
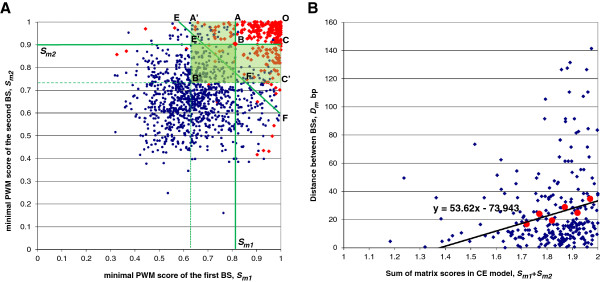
**Distributions of PWM scores and distances between BSs in real and random CEs. (A)** Distribution of PWM scores for first and second BSs in real CEs (red) and random sequence CEs (blue). Scores *S*_*m1*_ and *S*_*m2*_ define the rectangle OABC and perfectly separate high scoring CEs. By reducing the scores (dashed green lines), many additional true CEs, but also a large number of random CE are also covered by the rectangle OA′B′C′. Introduction of a sum of scores (diagonal EF) greatly improves the separation between real and random CEs (discontinuous line A′E′F′C′). **(B)** Distribution of distances between BSs and sum of matrix scores in real CEs (blue). Distance values were averaged in intervals of score values (1.75-1.80), (1.80-1.85), (1.85-1.90), (1.90-1.95) and (1.95-2.00) (red). The trend line reflects the dependence between PWM scores and distance between BSs.

It was observed that the combination “one BS with low PWM score – another with high PWM score” in real CEs is more frequent then “low - low” (distribution of PWM scores in the constructed CE models can be seen in Figure 1a). Pearson correlation coefficient calculated for PWM scores equals −0.164 (*p*-value 0.003) indicating negative correlation between matrix scores within one CE. To test the statistical relevance of this observation, we investigated the distribution of PWM scores (S_*m1*_, S_*m2*_) in matrix models of “random CEs”. Random sequence CEs were obtained from real CEs by reshuffling its DNA sequence. Matrix models for random CEs were constructed following the same procedure as for real CEs. The procedure with random CEs was repeated 4 times, generating 1060 models. Pearson correlation in this case was only −0.0088 (*p*-value 0.39).

Accuracy of the recognition method will obviously benefit when such mutual dependence of BSs is taken into account. From Figure 1A it becomes obvious that better separation of real and random CEs cannot be achieved by vertical or horizontal lines but rather by a diagonal. The diagonal corresponds to the sum of PWM scores, whereas vertical and horizontal lines are minimal scores for both BSs separately. Combination of restrictions on scores of both BSs individually (lines A′B′ and B′C′ on Figure 1A) and their sum (line EF) is one of the key points of the method and formally described in equation (4).

### Recognition rule

Mathematically this approach has to be described as follows. The diagonal or an absolute value of the composite score is defined by:

(1) $$\mathrm{absCS}=S_{m1}+S_{m2},$$

where S_*m1*_, S_*m2*_ are PWM scores defined by the CE model.

For the purpose of recognition we will use relative values for the composite score:

(2) $$\mathrm{relCS}=\frac{S_{m1}-S_{1}}{S_{m1}}+\frac{S_{m2}-S_{1}}{S_{2}},$$

where *S*_*1,2*_ are the actual matching scores of PWMs on an investigated DNA sequence. It is notable, that *relCS* may adopt negative values when one or both BSs of potential CE have higher PWM scores than defined by the model (S_*1*_ > S_*m1*_ and/or S_*2*_ > S_*m2*_). In such cases we say that the potential CE matches the model better than it is minimally required. Alternatively, another BS may have lower PWM score than required by the model, which corresponds to “high-low” phenomena described above.

To account for a relative positioning of BSs in CE we add a third term to (2):

(3) $$\mathrm{CS}=\frac{S_{m1}-S_{1}}{S_{m1}}+\frac{S_{m2}-S_{2}}{S_{m2}}+\lambda \left|D_{m}-D\right|,$$

where *D* is the actual distance between identified BSs and *D*_*m*_ - distance defined by CE model.

Considering the physics of DNA-protein and protein-protein interactions, it can be suggested that remotely located BSs both might have higher affinity to their TFs compared to closely located ones. Despite the fact that DNA may form loops and BSs distant by sequence may become close in 3D, we found this suggestion relevant and subjected it to verification.

Using all matrix models of CEs the distribution of distances between BSs (*D*_*m*_) and the *absCS* was calculated (Figure 1B). Averaged distance between BSs show that CEs that have longer distances between BSs have on average a higher *absCS*. Linear regression coefficient between distance and sum of scores equals 53.62 with a 90% confidence interval (40.9, 66.2). T-score of this regression is 7.6 with *p*-value of 0.004. 90% confidence interval for the slope value (53.62) equals (40.9, 66.2), 95% – (38.5, 68.7).Therefore, our assumption on dependence on distance and quality of BSs within a CE can be regarded as statistically relevant.

To make our method more stringent we considered both positive and negative fluctuation of distance *D* around the *D*_*m*_ as unfavorable. Coefficient *λ* in (3) was set to be equal to the slope value of the trend line (1/53.62).

Finally, a DNA sequence is reported as a potential CE, when the following recognition rule holds true:

(4) $$\left\{ \begin{array}{l} \frac{S_{m1}-S_{1}}{S_{m1}}\leq R_{1}\\ \frac{S_{m2}-S_{2}}{S_{m2}}\leq R_{2}\\ \;\mathrm{CS}\;\leq R_{\mathrm{CS}}\\ \frac{\left|D_{m}-D\right|}{D_{m}}\leq R_{D} \end{array},\right.$$

where *R*_*CS*_, *R*_*1*_, *R*_*2*_ and *R*_*D*_ are the relaxation parameters for the composite score *CS*, PWM scores and the distance respectively. A maximum stringency search is achieved with all these parameters set to 0.

### Input and output

To run MatrixCatch, the user should supply (a) DNA sequence(s) in EMBL, FASTA or plain text formats and (b) should define search stringency. Results are ordered by *p-*value. Threshold for *p*-value or expected frequency of CEs per 1kb can be optionally supplied. Calculation of raw *p-*values and its correction for multiple testing can be done using Bonferroni (5b), Bonferroni step-down (5c), and Benjamini and Hochberg (5d) procedures by the formulas:

(5a) $$p-\mathrm{value}=1-{\left(1-p\cdot q\right)}^{\left|D_{m}-D\right|}$$

(5b) $$\mathrm{corrected}\_p-\mathrm{valu}e^{B}=p-\mathrm{value}\cdot \mathrm{SequenceLength}$$

(5c) $$p-\mathrm{valu}e^{\mathrm{Bsd}}=p-\mathrm{value}\cdot \left(\mathrm{SequenceLength}-\mathrm{rank}\_\mathrm{CE}\right)$$

(5d) $$p-\mathrm{valu}e^{B\&H}=p-\mathrm{value}\cdot \left(\mathrm{SequenceLength}/\mathrm{rank}\_\mathrm{CE}\right),$$

where *p* (*q*) is a frequency of the first (second) BS of a CE found on a random sequence with PWM minimal score equals *S*_*1*_ (*S*_*2*_), and *rank_CE* is the rank of CE in the output list sorted by *p-*values before correction.

All *p*-value related parameters, namely *p*-value threshold, type of *p*-value correction or frequency of CEs per 1kb, can be adjusted after the search in order to refine the output. MatrixCatch produces a list of potential CEs, their positions, scores, *p*-values and respective links to the original CEs in the database. Graphical visualization and machine readable output is also provided.

In addition to the preloaded library users are encouraged to create, store and search for their own CE models (please visit the website). To do this a user should select PWMs from the existing library, specify thresholds, orientations, interspace distance and optionally give a description. Such an element of crowdsourcing allows a quick integration of novel data and its use by the community. A single composite regulatory element found in a specific experiment is already sufficient to be submitted into the system and used without a need for a programming and/or an establishment of a separate website. As a gratitude for such submissions, users who will use these models in their research are requested to cite the work of the submitter.

## Results

### Comparison with other CE recognition methods

At first, we compared our method to other available methods for CE prediction. CompelPatternSearch [6] is based on comparison of an original sequence of CE with an investigated sequence. By increasing the number of allowed nucleotide mismatches in both motifs and the distance between them the accuracy of the method can be adjusted. Another method was specifically developed for the recognition of composite element NF-AT/AP-1 [4] with a score function based on weighted logarithms of PWM scores and a fixed length of intermediate sequence from 5 to 11bp. False positive rates were estimated on sequences of second exons derived from the human genome, since they are supposed to comprise no regulatory elements. In all tests the elements to be recognized were excluded from the training data. All three methods were tested on the same dataset by the same procedure.

**Figure 2:**
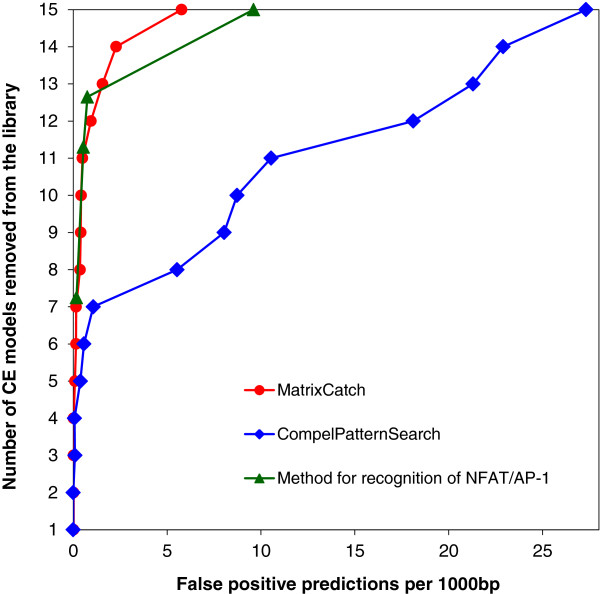
Receiver Operating Characteristic (ROC) curves of three methods on recognition of CE NFAT/AP-1.

Receiver operating characteristic (ROC) curves of the three methods tested on recognition of NF-AT/AP-1 are shown in Figure 2. ROC-curves for another two CEs (C/EBP/NFkappaB and E2F/Sp1) can be found in Additional file 1: Figures S1 and S2. These tests show that MatrixCatch in general outperforms the simple pattern based search used in CompelPatternSearch. CompelPatternSearch performs similarly only when used with most stringent parameters, i.e. when no mismatches are allowed in both BSs and length variation is not more than just a few nucleotides. Relaxing parameters results in a sharp increase of the false positive rate. Already with ≥2 allowed mismatches per BS, CompelPatternSearch becomes practically unusable due to extreme number of predictions (Additional file 1: Figure S1). MatrixCatch performance is much more tolerant to parameter relaxation. This also shows that MatrixCatch is less subjected to an over-training effect, since more knowledge is enclosed in CE matrix models rather than just in the DNA sequence of CE.

Unfortunately, many types of CEs are represented by a single example. In practical applications all are used for recognition, but for testing, obviously at least two known CEs of the same class are required. Therefore, a cross-validation for all elements is not feasible. We presented comparisons for two classes NF-AT/AP-1 and C/EBP/NFkappaB that have the highest number of examples. However, even for smaller classes the performance of MatrixCatch is evident (Additional file 1: Figures S2).

### Comparisons with statistical methods

First let us define what we call known, novel and *de novo* regulatory element. By known regulatory elements (both single sites and pairs) we assume those verified experimentally. By novel regulatory elements we assume those identified by any kind of computational comparison but without experimental verification on functionality. These elements can be found using similarity to known ones (then we say novel or potential BS and CE) or solely by statistical evaluations of motif frequencies in an investigated dataset (in this case we say *de novo* motif identification, for example see [1,10,11]). So, for example, MatrixCatch uses a library of CE models and hence finds novel composite elements. CMA and ModuleSearcher use a library for single sites (PWMs) and find novel single sites but discover pairs *de novo*. CisModule discovers single sites and pairs *de novo* purely based on statistics. Although these methods utilize different approaches, from practical view one would like to know which method(s) to apply first to, for example, a set of DNA sequences to have the highest chances of true discovery. In such cases collections of known elements are commonly used for evaluation of both library based and *de novo* methods.

For testing of the performance of MatrixCatch we selected well established benchmark datasets [1], and as a quality measure, we chose the nucleotide-level correlation coefficient (nCC). We preferred nCC over PPV (positive predictive value), since the latter did not accurately account for situations when, for example, a predicted module only slightly overlaps with a real one or is much longer then a real one. Instead nCC reflects the sensitivity and specificity of predictions by counting the number of correctly predicted nucleotides i.e. nucleotides that lie in an overlap of a predicted and a real module (for exact formula see [1]).

The selected benchmark consists of TRANSFAC matrices related to the composite elements to be identified, complemented by a number of “noise” matrices (not related to the CEs). Noise levels correspond to the number of the additional matrices in a set. The “noise_99” series comprises all PWMs. MatrixCatch was run with its default parameters, the entire library of CE models and with PWM datasets provided by [1] that correspond to the different noise levels. Reduction in the PWM library automatically directed MatrixCatch not to use CE models that comprise missing PWMs. Results obtained were submitted for evaluation (http://tare.medisin.ntnu.no/composite/composite.php). Unfortunately, coMOTIF [11] converged to equiprobable PWM (all elements equal 0.25) on all datasets. Other tests showed that coMOTIF performs better on data consisting from a large number of shorter sequences (data not shown).

**Figure 3:**
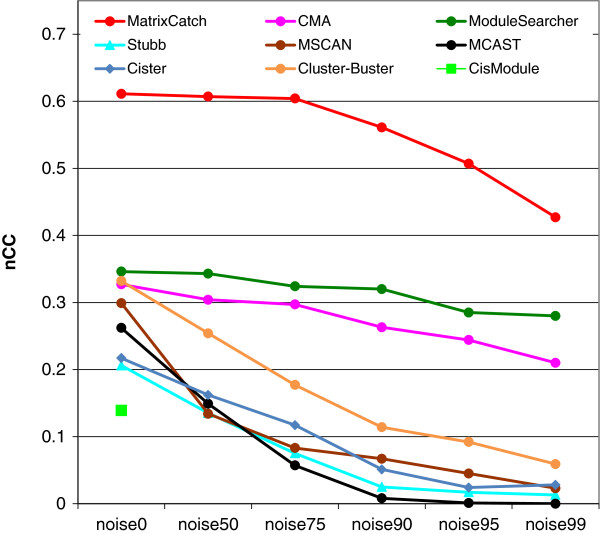
**Nucleotide level correlation scores (nCC) on the TRANSCompel dataset.** Nucleotide level correlation scores (nCC) on the TRANSCompel dataset. The graphs show nCC scores at increasing noise levels. Values for CisModule could be calculated only for the “noise0” dataset. For further details see (Klepper *et al*. [1]).

The results of the comparison are presented in Figure 3. It is evident that MatrixCatch significantly outperforms all other methods on all datasets. Despite such a good performance, one should note the different nature of these methods (*de novo* identification and library based) and the results need to be interpreted adequately.

MatrixCatch was used with the entire CE library. It identified all CEs in each of the datasets (data not shown), which would indicate a sensitivity of 100%. However, we should point out that the identified CEs are the same that were used to build the models and MatrixCatch by its definition always identifies the CEs used to construct the models. This is the major difference to comparisons in the previous section, where respective CE models were removed from the CE library. Thus, comparing the sensitivity parameter is not fully appropriate here.

Instead, specificities of the predictions should be compared. nCC score is calculated upon all reported CEs and its higher values in all categories for MatrixCatch indicate higher specificity. This can be interpreted in such a way that MatrixCatch not only identified all true CEs in the dataset but also did not report too many false hits.

However, if we assume that a dataset contains only regulatory elements principally different from those in the library, priority should be given to *de novo* identification methods. The practical application of MatrixCatch presented in the next section shows that the existing variety of known CEs is already sufficient to outperform statistical methods in most of situations.

### Investigation of tissue-specific promoters

An experimental study of tissue-specific promoters was recently performed by [12]. The authors investigated the expression of genes triggered by alternative promoters in different tissues. They could show that transcription from alternative promoters differs significantly in most investigated cases. Therefore, tissue specific promoters found in that study represent a competitive example for bioinformatics analysis. We will search for potential composite regulatory elements similar to known ones using MatrixCatch and novel combinations using other programs. The key question is which program can identify elements that are most specific to the dataset of interest.

Using the data provided by [12], 11 datasets of positive and negative promoters with a length of 500bp and 1kb that covered regions −400 to +100 and −900 to +100 around the TSS, respectively, were generated (datasets can be found in Additional file 2). For the discovery of *cis*-regulatory modules, methods reviewed by [1] were selected. Out of eight programs, two are not available to date (MSCAN and Stubb). Cluster-Buster and Cister could not be applied, since they require a single sequence as input, but not a set. MCAST identified very long modules with many motifs. For instance, in the 500bp breast dataset MCAST reported a module 355bp long with 23 motifs as a top hit. Though of very significant *E*-value, this result seems to have little practical use. Finally, only three programs, CisModule, ModuleSearcher and CMA in addition to MatrixCatch were used for the analysis.

The goal was to identify such a module(s) that can be found in at least *Min*^*+*^ of positive promoters and in no more than *Max*^*–*^ of the negative ones. If we denote *C*^*+*^ and *C*^*–*^ the normalized number of positive and negative promoters comprising a module, then the above can be formalized: *C*^*+*^ ≥ *Min*^*+*^ and *C*^*–*^ ≤ *Max*^*–*^. Several values for *Min*^*+*^ and *Max*^*–*^ were fixed: (0.90, 0.50), (0.75, 0.50), (0.66, 0.50), (0.50, 0.25), (0.33, 0.15).

All programs were run with default parameters except the following. The number of single PWMs in a module was set to 2 in CMA, ModuleSearcher and CisModule. In ModuleSearcher “Number of top scoring modules to return” was set to 10. CMA was set to output 5 pairs (maximum allowed) and to optimize distance of a module. Both above programs used the TRANSFAC library of PWMs. CisModule does not require PWMs, since it identifies them during the search. In summary, all programs were set to find several modules each consisting of a pair of DNA motifs. Since ModuleSearcher and CisModule cannot use negative datasets, the results of all three programs were additionally optimized in order to maximize the ratio *C*^*+*^/*C*^*–*^, provided that the boundary conditions for *C*^*+*^ and *C*^*–*^ hold true. This was done by varying independently the minimal required scores for both PWMs in a module and the one with the highest *C*^*+*^/*C*^*–*^ is reported as a hit. MatrixCatch was run with entire library of CE models and relaxation parameters were adjusted for maximum *C*^*+*^/*C*^*–*^.

We believe that this determination of the method performance is straightforward and is most indicative in real applications. Indeed, no common measures like false positives, true negatives *etc*. can be calculated, since regulatory modules are to be discovered *de novo*. Tests on re-discovery of known examples are presented above.

Results of the application of the four methods are presented in Tables 1, 2 and Additional file 1: Table S1. As can be seen from Table 1, in each specificity group MatrixCatch has found modules in more datasets, compared to the other methods. For example, in a group (*C*^*+*^ ≥ 0.75 and *C*^*–*^ ≤ 0.50) MatrixCatch found CEs in breast, heart, kidney and prostate promoters, while CMA and ModuleSearcher only in prostate promoters.

**Table 1:** recognition of regulatory elements in tissue specific promoters

**Specificity level (*Min***^***+ ***^ ***/ Max***^***– ***^ ***)***	**0.90/0.50**	**0.75/0.50**	**0.66/0.50**	**0.50/0.25**	**0.33/0.15**
MatrixCatch	1	4	7	4	5
CMA	0	1	3	0	1
ModuleSearcher	0	1	6	1	3
CisModule	0	0	1	1	2

Number of datasets of tissue specific promoters in which the programs found at least one module with the required level of specificity. The total number of datasets is 11.

**Table 2:** Specificity values of regulatory modules

**Dataset (number of seq.)**	**MatrixCatch**	**CMA**	**ModuleSearcher**	**CisModule**
Breast (24)	5.29	1.65	2.90	3.66
Heart (68)	2.60	–	1.38	–
Kidney (51)	3.47	1.46	2.54	–
Muscle (86)	1.43	–	1.35	–
Pancreas (61)	2.56	–	1.43	–
Prostate (17)	9.54	6.19	2.49	6.54
Thyroid (74)	1.62	–	1.40	–

Highest values of specificity (*C*^*+*^/*C*^*–*^) shown by the programs in different datasets. None of the programs found modules in the datasets: Cerebellum, Liver, Spleen and Testis.

Out of four methods only MatrixCatch was able to identify a regulatory element with very high specificity (group 0.90/0.50 in Table 1, CE number 112, relaxation parameters: *R*_*1*_=0.02, *R*_*2*_=0.26, *R*_*CS*_=0.20 and *R*_*D*_=0.32). This CE could be recognized in 16 out of 17 promoters active in prostate (*p*-value 5.624*10^-5^, promoters and CEs are graphically represented in Additional file 1: Figure S3). As was identified in a study of chicken myeloid cells both motifs of this CE are bound by C/EBP-related proteins [13]. It is very important to mention that C/EBP transcription factor was later found to upregulate metastatic gene expression in human prostate cancer cells [14,15]. This demonstrates that MatrixCatch identified highly specific regulatory elements the functionality of which was confirmed by several independent studies. In comparison, other programs could identify modules only in 13 (CMA, ModuleSearcher) or 12 (CisModule) promoters. None of the methods found an element similar to C/EBP binding motif. We may speculate that elements reported by statistical methods may represent some functionality, but no other support than statistical significance can yet be presented.

To emphasize the importance of the developed approach, we should mention that this type of CE is represented by a single example. As can be seen from Table 3 newly discovered CEs in prostate promoters don’t show many conserved positions in either motif. Approaches based on mere pattern matching of the DNA sequence of the CE itself (as for example, CompelPatternSearch [6]) would produce a huge number of hits, which renders predictions useless. Matching the motifs independently (as statistical methods do) will not help to reveal this CE either, due to the low score of one of the BSs. Indeed, composite elements in genes NET1, SULF1 MAD1L1, KIAA1539, SDR39U1 and COL4A6 have one C/EBP site recognized with a very low PWM score (Table 3). Nevertheless, the second site, recognized with a high PWM score, contributes to the overall composite score (3) of the pair. Thus, in all of the above-mentioned genes the composite score entailed specific recognition of the regulatory element.

**Table 3:** Composite element in prostate specific promoters

**Name**^**1**^	**Gene**	**Position**^**2**^	**Strand**	***S***_***1***_^**3**^	***S***_***2***_^**3**^	***CS***	***p*-value**	**Sequence**
Original composite element sequence								A**T**GA**GG**C**AAT** c*g*gcac*t* G**TT**GCCA**C**AT
uc002uum.1	MOB4	−346	+	0.972	0.976	0.012	3.801e-06	AGTT**T**GC**G**AA**AAT** gctg*tg* G**TT**TCTTAAGAGA
uc003jwu.1	OCLN	−213	+	0.973	0.949	0.202	9.234e-05	AGA**T**TCA**G**A**AA**CA gc*g*ccaa*tg* **TT**TACA**C**ACGACT
uc003qcg.1	EPB41L2	−100	+	0.988	0.981	0.099	1.418e-06	AGA**T**TTT**G**A**AAT**G ctac **TT**TTCA**C**AAAATA
uc001iia.1	NET1	−369	+	0.991	0.765	0.207	1.091e-05	ACC**T**TT**GG**T**AAT**T *g*gaaa*t* A**T**ATCT**C**ATATTG
uc002eby.1	ZNF843	−352	+	0.963	0.929	0.123	1.895e-04	AGCCTA**GG**C**AA**AA *g*agcac*g* A**TT**CCGT**C**TCAAG
uc004dpe.1	SHROOM4	+3	+	0.961	0.915	0.140	3.402e-04	TGC**T**ATT**G**T**AA**AT *g*gaac*tg* TT**TT**CTTT**C**TTTC
Sequence complementary to the original composite element sequence								A**TG**T**G**GC**A**AC *a*g*t*gccg A**TTGC**C**T**CAT
uc003edg.1	C3orf15	−317	–	0.946	0.959	0.110	1.347e-04	TGGC**TG**A**G**AA**A**AT c*aat*gac A**TTGC**T**T**ATGAAA
uc003fsb.1	TP63	−345	–	0.924	0.972	0.228	2.397e-04	ACAAA**G**A**G**TA**A**AA ag*aa*aag T**TT**T**C**A**T**AAAGGA
uc003gno.1	C1QTNF7	−27	–	0.947	0.997	0.234	4.040e-06	AAAC**TG**A**G**AA**A**GA t*aa* C**TT**T**C**TGAAATGC
uc003xye.1	SULF1	−333	–	0.728	0.987	0.304	1.456e-04	AAAGAAA**G**GT**A**GG c*a* G**TTGC**AAAACTTC
uc002tah.1	AFF3	−149	–	0.922	0.993	0.046	4.600e-06	TCAGAAG**G**AA**A**AA *a*g*t*ttag A**TT**T**C**AAAATGTA
uc003sli.1	MAD1L1	+2	–	0.761	0.981	0.276	1.790e-04	TGTC**T**AG**G**GG**A**GA t*aa*aat C**TTGC**C**T**AAGCAA
uc003zwl.1	KIAA1539	−310	–	0.760	0.959	0.300	8.385e-04	CTCCGTA**G**TC**A**CC agat*t*tt A**TT**T**C**ACAAGGTG
uc001lwy.1	SLC22A18	−113	–	0.939	0.965	0.167	1.691e-04	CGCTCCC**G**GA**A**CT tcc*at* T**TT**A**C**A**T**ATGAGG
uc001wpn.1	SDR39U1	−12	–	0.767	0.993	0.313	3.045e-05	TTAG**TG**A**G**AC**A**AT ggcg A**TTGC**AAAGCGCG
uc004env.1	COL4A6	−44	–	0.752	0.981	0.285	2.156e-04	TGAGATG**G**AC**A**TT ttat*t*ttt A**TTGC**C**T**AAACTG

Composite regulatory element C/EBP / C/EBP recognized in promoters of genes active in prostate tissue. Nucleotides with significant conservation shown in bold (within binding motifs) and italics (intermediate sequence).

^1^ Names according to (Jacox *et al.*, [12]).

^2^ Beginning of the element relative to TSS.

^3^*S*_*1,2*_ - PWM scores for the first and second C/EBP motif, *CS* - composite score.

Altogether, using the approach presented here it became possible to build up a matrix model for a singular example of a C/EBP/ C/EBP composite element and use this model for recognition of new potential regulatory elements in prostate promoters with high specificity. Therefore, highly reliable experimental knowledge is not dismissed due to statistical considerations.

We investigated potential composite elements identified with specificities C^+^ ≥ 0.75, C^–^ ≤ 0.50 (in Additional file 1: Table S1) for their biological relevance. CE NF-kappaB/ ATF-1 (relaxation parameters: *R*_*1*_=0.06, *R*_*2*_=0.10, *R*_*CS*_=0.70 and *R*_*D*_=0.48) was found specific (0.75/0.391) to promoters active in breast tissue and was described as activator of interleukin 2 gene [16]. Although neither NF-kappaB nor ATF-1 *per se* exhibits any specific tissue specificity, the NF-kappaB family has shown to be active in human breast cancers [17]. Taking into account that composite elements often have their own transcriptional function [8], this element may represent a promising example for further investigations. Another element c-Myb/Ets-1 (*R*_*1*_=0.08, *R*_*2*_=0.10, *R*_*CS*_=0.10 and *R*_*D*_=0.28), found in heart specific promoters, contains Ets-1 as one of the contributing factors, which has been shown to be expressed during heart development in mouse [18]. The third element HNF-4α/ HNF-4α found in kidney promoters (*R*_*1*_=0.20, *R*_*2*_=0.26, *R*_*CS*_=0.70 and *R*_*D*_=0.76) is known to play a role in development of the liver, kidney and intestines. Altogether, these examples show that MatrixCatch is able to identify potential composite elements that are not only specific, but are also biologically relevant to the investigated datasets. The biological knowledge behind is an important advantage in comparison to methods based on pure statistics.

An interesting dependence on the input data is shown by the programs CisModule and ModuleSearcher. ModuleSearcher identified regulatory modules substantially in 1kb promoters, whereas CisModule in 500bp (in Additional file 1: Table S1). Such a behavior may impede the practical applications of these methods since there is no agreement on a “proper” length of a promoter. MatrixCatch is more tolerant towards the input data as well as to the optimization of parameters. Results in Additional file 1: Table S1 show that in general MatrixCatch finds composite modules in many specificity groups. There are just a few cases when modules that discriminate positive and negative datasets are found exclusively in one specificity group which corresponds to one specific set of relaxation parameters. For example, modules found in pancreas and thyroid promoters are probably false hits, since they can be identified only in the specificity group (*C*^*+*^ ≥ 0.66, *C*^*–*^ ≤ 0.50), which may represent an artefact of parameters optimization. As a rule, if MatrixCatch identifies a composite module it can be found in several specificity groups, which proves greater tolerance to search parameters than in other methods.

## Discussion

Investigation of transcriptional regulation of genes by bioinformatic methods is widely used in biomedical research and the presented approach contributes to that topic. The software MatrixCatch is supplied with 265 matrix models of composite elements, which represents the most comprehensive collection of known CEs available to date. The program has no restriction on the size of promoters and is suitable for examination of a single short DNA locus of particular interest or big datasets representing the whole genomes. The search stringency can be easily adjusted via several parameters. The program was tested for recognition of known composite elements and compared with other programs on the established datasets. In all cases, MatrixCatch outperformed other methods. In a real study of tissue specific promoters, MatrixCatch identified a candidate composite element that is specific to promoters active in prostate, which we offer for further investigation. Other methods identified hits with much lower specificity and for many tissues they were not able to find any.

In the Introduction we pointed out that the problem in developing CE recognition methods lies in the extremely limited number of experimentally characterized and documented CEs. We may speculate that this could be a major reason why there is a bias towards statistical methods rather then methods based on experimental examples. In addition, many algorithms for the recognition of particular examples have no software implementation [3] or the announced web resource is not maintained anymore [5]. To the best of our knowledge, MatrixCatch is the only ready-to-use application available to date that is designed for recognition of known composite regulatory elements.

One fundamental question is whether DNA motifs constituting a CE and bound by interacting protein factors are similar to those bound by the same factors separately. This is an important issue, since it allows a generalization of the search by recruiting the information available for the single binding motifs. Similar performance of our method and the one described by [4] (Figure 2) suggests no or very minor changes of binding motifs, since the latter method uses exclusively DNA sequences of CEs for motif recognition. This method definitely accounts for all kinds of dependences between motifs - if any. But based on that principle, recognition methods could be constructed for just a few types of CEs, for 2 or 3 at best, since statistics become a critical issue. We can speculate that some TF binding motifs may be different in single sites and within composite elements, where they are bound by a TF complex. There are cases when subsets of a specific motif of single sites appear as constituents of CEs [19]. However, data available to date do not provide sufficient experimental evidences either to support or reject this. Similar results of this and the previous method [4] suggest that single binding motifs are at least not strongly changed, which allows to build a method for recognition of many types of CEs.

The presented approach has the advantage that already on the basis of any single identified CE, a matrix model can be constructed, which will ensure a reliable recognition. Thus, existing limited although valuable knowledge on combinatorial regulation of transcription can be used for the discovery of similar regulatory elements in other genes and/or related genes in different organisms. Together with other methods, both statistical and library based, MatrixCatch may serve as a basis for more sophisticated combinatorial analysis of promoters, enhancers or other regulatory regions, thereby helping to understand complex transcriptional regulation of genes and reconstruct complete hierarchical regulatory models.

## Conclusions

Here, we have presented a novel methodology for the identification of composite regulatory elements in promoter sequences. The software implementation MatrixCatch is supplied with a library of 265 matrix models used for recognition. That represents the widest scope of known CEs available to date. Additionally, this library can be easily extended via user supplied models. Investigation of regularities encoded in known composite elements helped to improve the specificity of the identification compared to other methods, that is proved on an established benchmark and real genomic data. Another advantage of the approach is that on the basis of any single newly discovered CE, a matrix model can be constructed and used for the recognition. A practical advantage of this method compared to statistical methods is the direct biological interpretation of the identified results.

## Competing interests

The authors declare that they have no competing interests.

## Authors’ contributions

IVD: implementation of MatrixCatch, construction of matrix models, writing the ms; OVK: initial data management, expert advice on structure of CE; EVD: critical comments on the program and the ms; AEK, EW: conceptual idea for MatrixCatch and writing the ms, SW project coordinator and writing the ms. All authors read and approved the final manuscript.

## Supplementary Material

Additional file 1 Supplementary Figures and Tables. Click here for file

Additional file 2 Dataset of tissue specific promoters. Click here for file
