# Supplementary material for: MatrixCatch - a novel tool for the recognition of composite regulatory elements in promoters
Source: BMC Bioinformatics. 2013 Aug 8;14:241. doi: 10.1186/1471-2105-14-241 (PMC3754795; doi:10.1186/1471-2105-14-241)

Supplementary Material for the article

MatrixCatch - a novel tool for the recognition of composite regulatory elements in promoters

Igor V. Deyneko[1*], Alexander E. Kel[2,3], Olga V. Kel-Margoulis[2], Elena V. Deineko[4], Edgar Wingender[2,5], Siegfried Weiss[1]

[1]Department of molecular immunology, Helmholtz Centre for Infection Research, Braunschweig, Germany

[2]GeneXplain GmbH, Wolfenbüttel, Germany

[3]Institute of Chemical Biology and Fundamental Medicine SB RAS, Novosibirsk, Russia;

[4]Laboratory of Plant Bioengineering, Institute of Cytology and Genetics SB RAS, Novosibirsk, Russia

[5]Department of Bioinformatics, Medical School, Georg August University of Göttingen, Göttingen, Germany

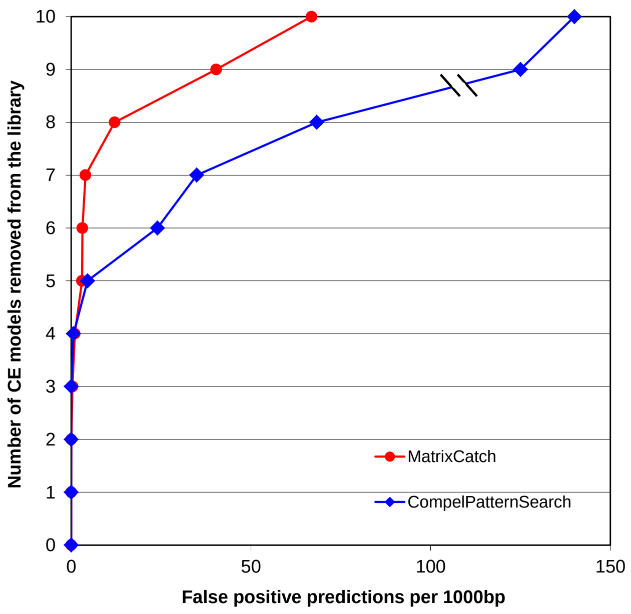

Fig. S1. Receiver Operating Characteristic (ROC) curves of the methods on recognition of CE CEBP/NFkappaB. This CE has 11 known examples. Two last points correspond to 2 and 3 allowed mismatches per BS. This demonstrates that pattern-based methods like CompelPatternSearch are efficient only for searches with very rigorous parameters.

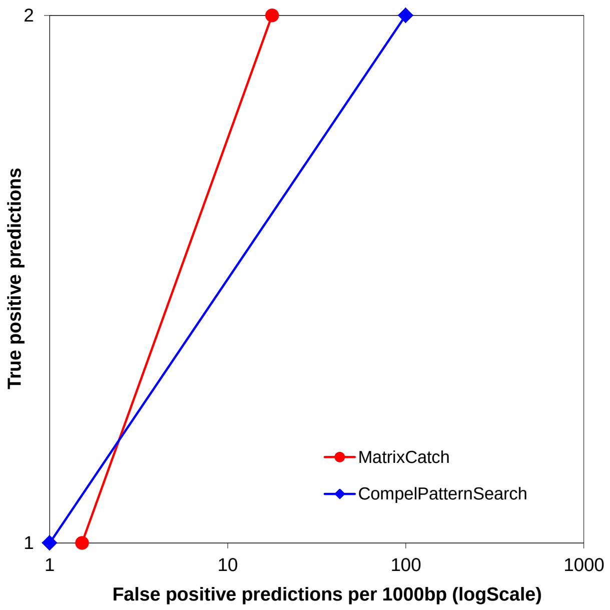

Fig. S2. Receiver Operating Characteristic (ROC) curves of the methods on recognition of CE E2F/Sp1. This CE has 2 known examples.

Table S1. Identification of regulatory modules in tissue specific promoters using several methods

MatrixCatch

500bp	090_050			075_050			066_050			050_025			033_015		
	C^+	C^-	ratio	C^+	C^-	ratio	C^+	C^-	ratio	C^+	C^-	ratio	C^+	C^-	ratio
Breast	-	-	-	0.750	0.391	**1.918**	0.667	0.279	**2.391**	0.500	0.168	**2.976**	0.333	0.063	**5.286**
Cerebellum	-	-	-	-	-	-	-	-	-	-	-	-	-	-	-
Heart	-	-	-	0.765	0.496	**1.542**	0.662	0.382	**1.733**	0.500	0.239	**2.092**	0.338	0.141	**2.397**
Kidney	-	-	-	0.765	0.479	**1.597**	0.667	0.372	**1.793**	0.529	0.235	**2.251**	0.333	0.096	**3.469**
Liver	-	-	-	-	-	-	-	-	-	-	-	-	-	-	-
Muscle	-	-	-	-	-	-	-	-	-	-	-	-	-	-	-
Pancreas	-	-	-	-	-	-	0.672	0.402	**1.672**	-	-	-	0.344	0.145	**2.372**
Prostate	0.941	0.489	**1.924**	0.765	0.305	**2.508**	0.706	0.254	**2.780**	0.529	0.071	**7.451**	0.353	0.037	**9.541**
Spleen	-	-	-	-	-	-	-	-	-	-	-	-	-	-	-
Testes	-	-	-	-	-	-	-	-	-	-	-	-	-	-	-
Thyroid	-	-	-	-	-	-	0.662	0.410	**1.615**	-	-	-	-	-	-

1Kb	090_050			075_050			066_050			050_025			033_015		
	C^+	C^-	ratio	C^+	C^-	ratio	C^+	C^-	ratio	C^+	C^-	ratio	C^+	C^-	ratio
Breast	-	-	-	0.750	0.359	**2.089**	0.667	0.260	**2.565**	0.500	0.167	**2.994**	0.333	0.072	**4.625**
Cerebellum	-	-	-	-	-	-	-	-	-	-	-	-	-	-	-
Heart	-	-	-	0.750	0.472	**1.589**	0.691	0.417	**1.657**	-	-	-	0.338	0.130	**2.600**
Kidney	-	-	-	0.804	0.495	**1.624**	0.667	0.368	**1.813**	-	-	-	0.333	0.101	**3.297**
Liver	-	-	-	-	-	-	-	-	-	-	-	-	-	-	-
Muscle	-	-	-	-	-	-	0.686	0.481	**1.426**	-	-	-	-	-	-
Pancreas	-	-	-	-	-	-	0.672	0.399	**1.684**	0.508	0.233	**2.180**	0.361	0.141	**2.560**
Prostate	0.941	0.442	**2.129/1**	0.765	0.328	**2.332**	0.706	0.252	**2.802**	0.529	0.107	**4.944**	0.412	0.056	**7.357**
Spleen	-	-	-	-	-	-	-	-	-	-	-	-	-	-	-
Testes	-	-	-	-	-	-	-	-	-	-	-	-	-	-	-
Thyroid	-	-	-	-	-	-	0.662	0.475	**1.394**	-	-	-	-	-	-

CMA

500bp	090_050			075_050			066_050			050_025			033_015		
	C^+	C^-	ratio	C^+	C^-	ratio	C^+	C^-	ratio	C^+	C^-	ratio	C^+	C^-	ratio
Breast	-	-	-	-	-	-	0.667	0.491	**1.358**	-	-	-	-	-	-
Cerebellum	-	-	-	-	-	-	-	-	-	-	-	-	-	-	-
Heart	-	-	-	-	-	-	-	-	-	-	-	-	-	-	-
Kidney	-	-	-	-	-	-	0.667	0.456	**1.463**	-	-	-	-	-	-
Liver	-	-	-	-	-	-	-	-	-	-	-	-	-	-	-
Muscle	-	-	-	-	-	-	-	-	-	-	-	-	-	-	-
Pancreas	-	-	-	-	-	-	-	-	-	-	-	-	-	-	-
Prostate	-	-	-	0.765	0.408	**1.875**	0.765	0.408	**1.875**	-	-	-	0.353	0.096	**3.677**
Spleen	-	-	-	-	-	-	-	-	-	-	-	-	-	-	-
Testes	-	-	-	-	-	-	-	-	-	-	-	-	-	-	-
Thyroid	-	-	-	-	-	-	-	-	-	-	-	-	-	-	-

1Kb	090_050			075_050			066_050			050_025			033_015		
	C^+	C^-	ratio	C^+	C^-	ratio	C^+	C^-	ratio	C^+	C^-	ratio	C^+	C^-	ratio
Breast	-	-	-	-	-	-	0.667	0.405	**1.647**	-	-	-	-	-	-
Cerebellum	-	-	-	-	-	-	-	-	-	-	-	-	-	-	-
Heart	-	-	-	-	-	-	-	-	-	-	-	-	-	-	-
Kidney	-	-	-	-	-	-	0.706	0.495	**1.426**	-	-	-	-	-	-
Liver	-	-	-	-	-	-	-	-	-	-	-	-	-	-	-
Muscle	-	-	-	-	-	-	-	-	-	-	-	-	-	-	-
Pancreas	-	-	-	-	-	-	-	-	-	-	-	-	-	-	-
Prostate	-	-	-	0.765	0.485	**1.577**	0.765	0.485	**1.577**	-	-	-	0.353	0.057	**6.193**
Spleen	-	-	-	-	-	-	-	-	-	-	-	-	-	-	-
Testes	-	-	-	-	-	-	-	-	-	-	-	-	-	-	-
Thyroid	-	-	-	-	-	-	-	-	-	-	-	-	-	-	-

Table S1. (continued)

ModuleSearcher

**500bp**	**090_050**			**075_050**			**066_050**			**050_025**			**033_015**		
	C^+	C^-	ratio	C^+	C^-	ratio	C^+	C^-	ratio	C^+	C^-	ratio	C^+	C^-	ratio
Breast	-	-	-	-	-	-	0.667	0.467	**1.428**	-	-	-	-	-	-
Cerebellum	-	-	-	-	-	-	-	-	-	-	-	-	-	-	-
Heart	-	-	-	-	-	-	-	-	-	-	-	-	-	-	-
Kidney	-	-	-	-	-	-	0.725	0.479	**1.514**	-	-	-	0.333	0.131	**2.542**
Liver	-	-	-	-	-	-	-	-	-	-	-	-	-	-	-
Muscle	-	-	-	-	-	-	0.674	0.499	**1.351**	-	-	-	-	-	-
Pancreas	-	-	-	-	-	-	-	-	-	-	-	-	-	-	-
Prostate	-	-	-	-	-	-	0.706	0.418	**1.689**	-	-	-	-	-	-
Spleen	-	-	-	-	-	-	-	-	-	-	-	-	-	-	-
Testes	-	-	-	-	-	-	-	-	-	-	-	-	-	-	-
Thyroid	-	-	-	-	-	-	0.662	0.498	**1.329**	-	-	-	-	-	-

**1Kb**	**090_050**			**075_050**			**066_050**			**050_025**			**033_015**		
	C^+	C^-	ratio	C^+	C^-	ratio	C^+	C^-	ratio	C^+	C^-	ratio	C^+	C^-	ratio
Breast	-	-	-	-	-	-	0.708	0.416	**1.702**	0.500	0.241	**2.075**	0.417	0.144	**2.896**
**Cerebellum**	-	-	-	-	-	-	-	-	-	-	-	-	-	-	-
Heart	-	-	-	-	-	-	0.662	0.479	**1.382**	-	-	-	-	-	-
Kidney	-	-	-	-	-	-	0.686	0.436	**1.573**	-	-	-	0.333	0.148	**2.250**
**Liver**	-	-	-	-	-	-	-	-	-	-	-	-	-	-	-
Muscle	-	-	-	-	-	-	-	-	-	-	-	-	-	-	-
Pancreas	-	-	-	-	-	-	0.672	0.471	**1.427**	-	-	-	-	-	-
Prostate	-	-	-	0.765	0.440	**1.739**	0.765	0.440	**1.739**	-	-	-	0.353	0.142	**2.486**
**Spleen**	-	-	-	-	-	-	-	-	-	-	-	-	-	-	-
**Testes**	-	-	-	-	-	-	-	-	-	-	-	-	-	-	-
Thyroid	-	-	-	-	-	-	0.662	0.474	**1.397**	-	-	-	-	-	-

CisModule

**500bp**	**090_050**			**075_050**			**066_050**			**050_025**			**033_015**		
	C^+	C^-	ratio	C^+	C^-	ratio	C^+	C^-	ratio	C^+	C^-	ratio	C^+	C^-	ratio
Breast	-	-	-	-	-	-	-	-	-	-	-	-	0.333	0.091	**3.659**
Cerebellum	-	-	-	-	-	-	-	-	-	-	-	-	-	-	-
Heart	-	-	-	-	-	-	-	-	-	-	-	-	-	-	-
Kidney	-	-	-	-	-	-	-	-	-	-	-	-	-	-	-
Liver	-	-	-	-	-	-	-	-	-	-	-	-	-	-	-
Muscle	-	-	-	-	-	-	-	-	-	-	-	-	-	-	-
Pancreas	-	-	-	-	-	-	-	-	-	-	-	-	-	-	-
Prostate	-	-	-	-	-	-	0.706	0.497	**1.421**	0.529	0.239	**2.213**	0.353	0.054	**6.537**
Spleen	-	-	-	-	-	-	-	-	-	-	-	-	-	-	-
Testes	-	-	-	-	-	-	-	-	-	-	-	-	-	-	-
Thyroid	-	-	-	-	-	-	-	-	-	-	-	-	-	-	-

**1Kb**	**090_050**			**075_050**			**066_050**			**050_025**			**033_015**		
	C^+	C^-	ratio	C^+	C^-	ratio	C^+	C^-	ratio	C^+	C^-	ratio	C^+	C^-	ratio
Breast	-	-	-	-	-	-	-	-	-	-	-	-	0.375	0.139	**2.698**
Cerebellum	-	-	-	-	-	-	-	-	-	-	-	-	-	-	-
Heart	-	-	-	-	-	-	-	-	-	-	-	-	-	-	-
Kidney	-	-	-	-	-	-	-	-	-	-	-	-	-	-	-
Liver	-	-	-	-	-	-	-	-	-	-	-	-	-	-	-
Muscle	-	-	-	-	-	-	-	-	-	-	-	-	-	-	-
Pancreas	-	-	-	-	-	-	-	-	-	-	-	-	-	-	-
Prostate	-	-	-	-	-	-	-	-	-	-	-	-	-	-	-
Spleen	-	-	-	-	-	-	-	-	-	-	-	-	-	-	-
Testes	-	-	-	-	-	-	-	-	-	-	-	-	-	-	-
Thyroid	-	-	-	-	-	-	-	-	-	-	-	-	-	-	-

Fig. S3. Graphical representation of composite elements and modules found by different programs. In cases when two or more modules found by the same program overlap, only the most significant module is shown. CMA and ModuleSearcher both selected EVI1 motif as a part of a module, but used different PWMs for it and selected different second motif. This may be a reason for low correlation between modules found by CMA and ModuleSearcher. In general, no correlation between all programs could be observed. Since ModuleSearcher failed to identify any modules in 500bp promoter sets in the specificity group (0.75, 0.50), modules found by the program for 1kb promoters is shown here when possible.

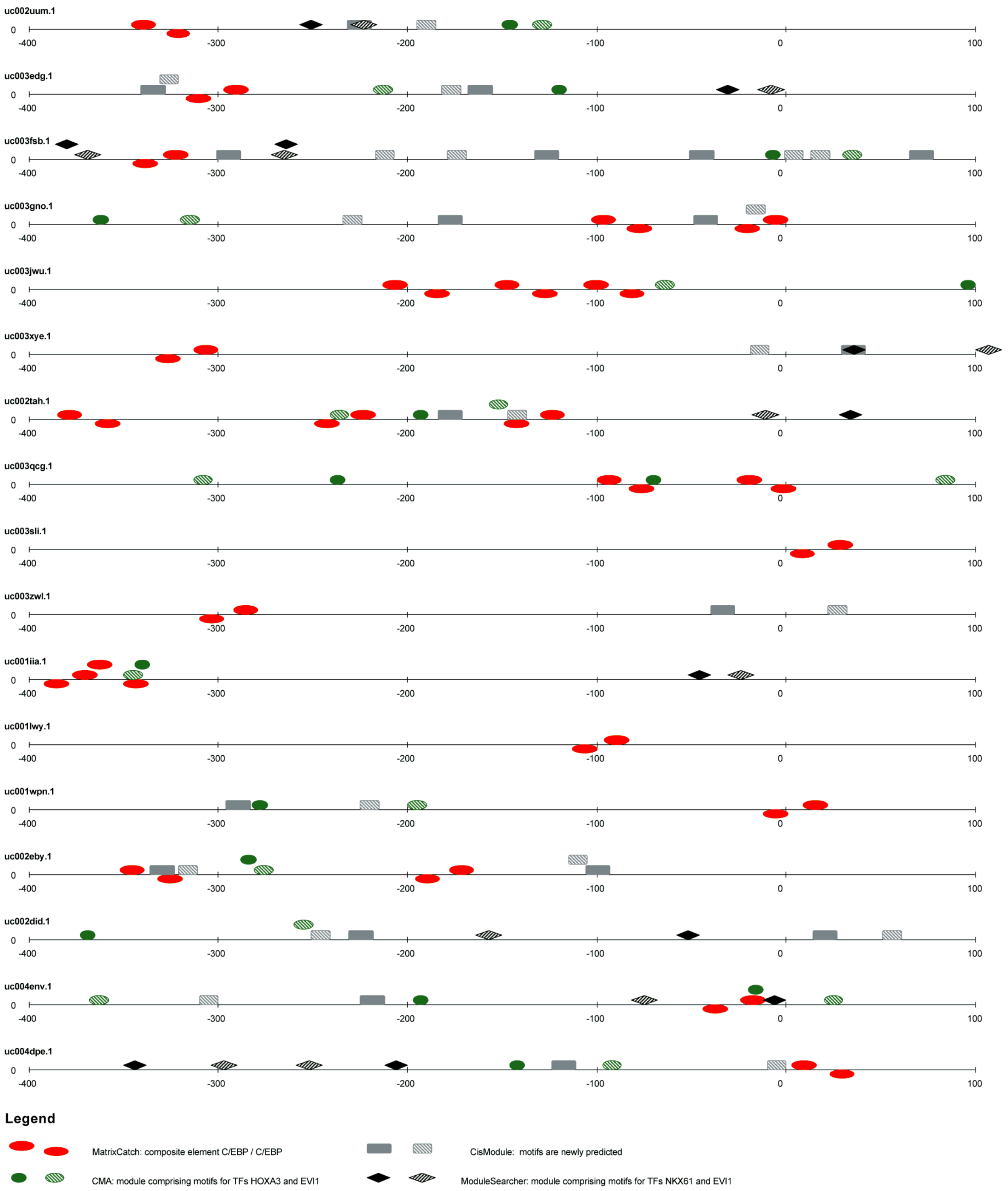

Supplement: Additional file 1 — Supplementary Figures and Tables. [file 1471-2105-14-241-S1.pdf]
